# Supplementary material for: Association between bicarbonate levels and mortality among acute respiratory distress syndrome patients: An analysis based on Medical Information Mart for Intensive Care database
Source: PLoS One. 2025 Jun 10;20(6):e0325498. doi: 10.1371/journal.pone.0325498 (PMC12151428; doi:10.1371/journal.pone.0325498)
Supplement: S1 Table — (DOCX) [file pone.0325498.s001.docx]

**Table S1 Sensitivity analysis results before and after imputations**

| Variables | Before imputation (N=6377) | After imputation (N=6377) | Statistics | *P* |
| --- | --- | --- | --- | --- |
| Systolic blood pressure, Mean (±SD) | 120.80 (±23.36) | 120.80 (±23.36) | t = 0.001 | 1.000 |
| SpO_2_, Mean (±SD) | 97.80 (±3.01) | 97.80 (±3.01) | t = 0.008 | 0.994 |
| PH, Mean (±SD) | 7.35 (±0.10) | 7.35 (±0.10) | t = -0.006 | 0.995 |
| Glucose, M (Q₁, Q₃) | 141 (116-175) | 141 (116-175) | W = 20329398 | 0.998 |
| Heart rate, Mean (±SD) | 88.87 (±19.58) | 88.87 (±19.58) | t = -0.009 | 0.992 |
| Diastolic blood pressure, Mean (±SD) | 65.81 (±15.87) | 65.81 (±15.87) | t = -0.005 | 0.996 |
| Urine output 24h, M (Q₁, Q₃) | 1705 (1131-2420) | 1705 (1132-2420) | W = 20313862.5 | 0.976 |
| Platelet, Mean (±SD) | 201.15 (±109.17) | 201.17 (±109.08) | t = -0.012 | 0.991 |
| Hemoglobin, Mean (±SD) | 10.77 (±2.19) | 10.77 (±2.19) | t = 0.002 | 0.999 |
| WBC, M (Q₁, Q₃) | 12.4 (8.8-16.6) | 12.4 (8.8-16.6) | W = 20286105.5 | 0.984 |
| Temperature, Mean (±SD) | 36.64 (±0.76) | 36.63 (±0.75) | t = 0.807 | 0.420 |
| Magnesium, Mean (±SD) | 2.03 (±0.51) | 2.04 (±0.50) | t = -0.831 | 0.406 |
| PT, M (Q₁, Q₃) | 14.4 (12.9-16.3) | 14.5 (13-16.3) | W = 18206922 | 0.356 |
| INR, M (Q₁, Q₃) | 1.3 (1.2-1.5) | 1.3 (1.2-1.5) | W = 18188124 | 0.328 |
| Lactate, M (Q₁, Q₃) | 1.9 (1.3-2.8) | 1.9 (1.4-2.7) | W = 18325584 | 0.710 |
| Respiratory rate, Mean (±SD) | 18.56 (±5.85) | 18.44 (±5.64) | t' = 1.146 | 0.252 |
| Weight, Mean (±SD) | 86.07 (±21.95) | 86.07 (±20.94) | t' = 0.004 | 0.997 |
| Phosphate, M (Q₁, Q₃) | 3.5 (2.9-4.3) | 3.5 (3-4.2) | W = 17323871.5 | 0.962 |
| Calcium, M (Q₁, Q₃) | 8.1 (7.7-8.6) | 8.1 (7.7-8.5) | W = 16944894.5 | 0.142 |

Notes: SpO_2_: oxygen saturation; WBC: white blood cell count; PT: prothrombin time; INR: international normalized ratio; SD: Standard Deviation; M: Median; Q₁: 1st Quartile; Q₃: 3st Quartile; t: Student's t test; t': Satterthwaite t test; W: Wilcoxon rank sum test.
