# Supplementary material for: Association between bicarbonate levels and mortality among acute respiratory distress syndrome patients: An analysis based on Medical Information Mart for Intensive Care database
Source: PLoS One. 2025 Jun 10;20(6):e0325498. doi: 10.1371/journal.pone.0325498 (PMC12151428; doi:10.1371/journal.pone.0325498)
Supplement: Attachment — Submitted filename: Responses to reviewers.docx [file pone.0325498.s003.docx]

**Introduction:**

- While the term endothelial dysfunction is introduced in line 55, a brief explanation of what it entails, specifically in the context of ARDS.

Reply: Thanks for your comment. In describing the pathological basis of patients with ARDS, we further describe the association of endothelial dysfunction with the occurrence and progression of ARDS: Previous clinical studies have revealed that ARDS is characterized by inflammatory pulmonary edema and impaired gas exchange, with endothelial injury serving as one of the pivotal mechanisms underlying its pathogenesis and progression. Endothelial injury drives the pathological process of ARDS by increasing microvascular permeability, disrupting the coagulation-fibrinolysis balance, and amplified inflammatory responses.

- In lines 56-61, you mention that low bicarbonate levels reflect metabolic acidosis and that this is a contributing factor to poor outcomes in ARDS patients. Consider specifying how bicarbonate directly impacts acid-base balance and how metabolic acidosis worsens ARDS.

Reply: Thanks for your comment. We systematically elucidated the association between bicarbonate levels and systemic acid-base balance, along with the specific pathways through which metabolic acidosis exacerbates ARDS：The bicarbonate buffer system serves as one of the most primary buffer systems for maintaining acid-base balance in the human body. When acidic substances increase, bicarbonate can combine with hydrogen ions to form carbon dioxide and water, thereby neutralizing the acids. Excessive loss bicarbonate compromises the body’s ability to neutralize acid, leading to accumulation of acidic substances in extracellular fluid and subsequent metabolic acidosis. Metabolic acidosis aggravates ARDS by disrupting the alveolar-capillary barrier, increasing endothelial injury and amplifying inflammatory responses

- emphasize the novelty of the study a bit more.

Reply: Thanks for your comment. We have elaborated on the novelty and clinical significance of this article in the Introduction and Discussion sections: Understanding this relationship may provide valuable insights into risk stratification and the potential role of bicarbonate levels as a prognostic marker, contributing to more clinical decision-making and tailored management strategies for ARDS patients.

- The objective of the study is well-stated, but could be more precise in how it will help clinical decision-making

Reply: Thanks for your comment. In the introduction section, we briefly described the potential clinical significance of this study. In the discussion part, we elaborated in detail on the clinical significance of using baseline bicarbonate level as a potential prognostic indicator for patients with ARDS.

Baseline bicarbonate level, as a potential prognostic biomarker for ARDS, holds dual clinical significance: on the one hand, lower bicarbonate levels reflect the metabolic acidosis and is associated with multi-organ dysfunction; on the other hand, lower bicarbonate levels can provide a reference for the stratification of mortality risk and help guide more effective clinical management of patients with ARDS in the ICU.

**Methods:**

- Add s brief note on the validation of the MIMIC-IV database or any potential limitations specific to the dataset that might affect the study's conclusions.

Reply: Thanks for your comment. We have added the verification information for the MIMIC- IV database: To access this database, one of the authors (Junli Han) obtained the necessary certification and subsequently extracted the relevant variables for our study (certification number: 13804349). We have elaborated on the limitations of the MIMIC-IV database in the discussion section.

- Expand to specify how the data was structured (e.g., patient demographics, medical histories, treatment details)

Reply: Thanks for your comment. In the data extraction section of the manuscript, we have presented the clinical data extracted in this study in detail by category such as demographic information, comorbidities, vital signs, and treatment modalities. Furthermore, we have also re-presented the baseline table in accordance with such categories.

- The statement "ethical approval from the Second Affiliated Hospital of Xi'an Jiaotong University was waived" needs additional clarification.

Reply: Thanks for your comment. We have re-stated the ethical approval for this study: The use of the MIMIC-IV database was approved by the review committee of Massachusetts Institute of Technology and Beth Israel Deaconess Medical Center， therefore, the ethics review committee of the Second Affiliated Hospital of Xi’an Jiaotong University waived the ethics approval statement and the requirement for informed consent for this study.

- The term "meticulously" in "meticulously extracted" could be omitted or replaced with more objective language, as "meticulously" is somewhat subjective. Consider using "systematically" or "using predefined codes."

Reply: Thanks for your comment. We have revised “meticulously” to “systematically”.

- Briefly mention the specific criteria used in the Berlin definition of ARDS.

Reply: Thanks for your comment. We have already described the Berlin definition of ARDS and further described the definitions of mild, moderate and severe ARDS: ARDS was defined according to the Berlin criteria, which include the following: acute onset, a PaO2/FiO2 ≤300 mmHg, PEEP ≥5 cm H2O, bilateral infiltrates on chest radiograph, and the absence of heart failure. ARDS severity was classified based on the PaO2/FiO2 ratio: mild (200 mmHg <PaO2/FiO2 ≤300 mmHg), moderate (100 mmHg <PaO2/FiO2 ≤200 mmHg), and severe (PaO2/FiO2 ≤100 mmHg).

- Clarify the purpose of each statistical model and analysis, especially the difference between univariate and multivariable Cox models.

Reply: Thanks for your comment. We have described in detail the purpose of implementing each model and analysis in the statistical analysis section. Among them, the univariate Cox hazard proportional model was used to preliminarily screen the covariates with statistical association with the 28-day mortality risk of ARDS patients, excluding non-statistically significant variables, and reducing the dimension of subsequent multivariate analysis. The covariates with statistically significant associations with outcomes selected from the univariate Cox proportional hazards model analysis were included in the multivariate Cox proportional hazards model analysis, and the association between bicarbonate levels and the 28-day mortality risk of ARDS patients was clarified by controlling for confounding.

- Expand on how sensitivity analysis was performed and why it was necessary, especially the comparison between datasets before and after imputation.

Reply: Thanks for your comment. Sensitivity analysis of missing data before and after imputation is a critical step in clinical research to ensure the reliability of results. We excluded variables with missing value >20%, and variables with missing value ≤20% were multi-imputed. In our study, we describe in detail the method of multiple imputation, and the purpose of the sensitivity analysis before and after multiple imputation: that is, considering the potential impact of this multiple imputation method on the relationship between exposure and outcome, we performed a sensitivity analysis comparing the results before and after multiple imputation to confirm the robustness of the model and exclude the effect of random forest imputation on the results.

- The statement "detailed results are presented in Supplementary Table 1" need more context about the importance of the table or a brief description of what it contains to guide readers.

Reply: Thanks for your comment. Supplementary Table 1 presented the sensitivity analysis before and after the imputation of missing data. We elaborated on the results of Supplementary Table 1 in the manuscript: The distribution differences of all variables before and after imputation were not statistically significant (all P>0.05), thus, the imputation of missing data conducted in this study supported the robustness of the study results.

- The description of the multiple imputation process is generally clear; however, the reference or package name is missing after "mice()" — please insert the appropriate package name or a proper citation inside the parentheses.

Reply: Thanks for your comment. The parentheses indicate this is the standard function call syntax in R programming, where:

- mice refer to the Multiple Imputation by Chained Equations algorithm;
- Empty parentheses () denote the function's default parameter structure

We have made the following revisions in the manuscript：Missing values were handled using the mice () function from R’s mice package (v 4.3.1), which starts with a data frame containing missing values and generates multiple (default of five) complete datasets by imputing the missing values.

- The choice of the 65-year cutoff for subgrouping by age should be justified, either by citing clinical relevance or previous literature.

Reply: Thanks for your comment. To assess the robustness of the association between baseline bicarbonate levels and 28-day mortality risk in ARDS patients, we stratified the study population into two subgroups: those **aged ≥65 years** and those **<65 years**. The rationale for selecting **65 years as the cutoff** was based on the following considerations: **first, from the perspective of biological Plausibility,** 65 years is a well-established critical threshold for "immunosenescence," characterized by significant decline in alveolar epithelial repair capacity and more severe dysregulation of inflammatory responses; second, The Berlin Definition classifies **older ARDS patients as those aged ≥65 years**, reflecting distinct pathophysiological and prognostic features in this population; finally, in the U.S., **65 years marks the eligibility age for Medicare coverage**, which may influence treatment intensity decisions in clinical practice.

In summary, we conducted stratified analyses by age (≥65 vs. <65 years) to further investigate the relationship between baseline bicarbonate levels and 28-day mortality risk across different age groups in ARDS patients.

**Discussion**

- The discussion presents a large number of studies with repetition of the main finding several times (e.g., lines 268–272, 326–328, 342–344, 384–386) back-to-back without a strong thematic integration. This can overwhelm the reader and obscure your main findings. Group the studies thematically: For example, start by discussing studies on bicarbonate levels in non-ARDS populations (e.g., cancer, dialysis, stroke), then transition into critically ill populations, and finally position your study within this context. Use linking sentences between studies to explain how each cited work builds toward the necessity of your study.

Reply: Thanks for your comment. Thank you very much for your valuable suggestions. We have reorganized the discussion part in accordance with your opinions. All the revisions are presented in red marks in the discussion section of the manuscript.

- There’s a very long list of studies about bicarbonate levels in different diseases (lines 273–313), but only some of them are truly relevant to ARDS. The link to ARDS literature is weak. Most cited studies are on *CKD*, *dialysis*, *cancer*, or *stroke* patients, not ARDS patients.

Reply: Thanks for your comment. Current clinical research on the association between bicarbonate levels and short-term prognosis in ARDS patients remains limited. In our discussion section, we initially reviewed existing evidence regarding bicarbonate levels and outcomes in various diseases. This approach served two purposes:

1. It highlighted the significance and novelty of our study by contextualizing it within the substantial disease burden of ARDS;
2. It provided clinical research references for investigating the bicarbonate-ARDS prognosis relationship.

Subsequently, we focused on explaining the potential physiological mechanisms underlying the association between bicarbonate levels and short-term outcomes in ARDS patients. In response to your valuable suggestion, we have substantially reduced the discussion of bicarbonate's role in other diseases, shifting our emphasis to interpreting the specific findings of our current study.

- Bicarbonate treatment is mentioned in subgroups but is not deeply discussed. Briefly discuss whether bicarbonate supplementation has clear evidence in ARDS treatment or remains controversial.

Reply: Thanks for your comment. We conducted subgroup analyses of patients with ARDS with different characteristics, and in the discussion, we attempted to explain the results of the subgroups in terms of physiological mechanisms. However, the focus of our study was to explore the association between baseline bicarbonate levels and the 28-day mortality risk in patients with ARDS, and the aim of subgroup analyses was to demonstrate that this association remained robust in patients with ARDS with different characteristics, and to increase some confidence in the association between exposure and outcomes. We also stated in the article that the association between baseline bicarbonate levels and short-term outcomes in patients with ARDS with different characteristics still needs further specialized studies to unravel. In addition, we looked at the association between baseline bicarbonate levels and their short-term mortality risk in patients with ARDS admitted to the ICU, rather than the association between bicarbonate use and their prognosis. Of course, the association between bicarbonate supplementation and the prognosis of ARDS patients is a very valuable research direction, and I hope that we or other researchers can further pay attention to the association between bicarbonate use and the prognosis of ARDS patients in the future, to provide more insights for clinicians to carry out accurate medical management of ARDS patients.

- You mention that this is the first study to address bicarbonate and 28-day mortality in ARDS patients using the MIMIC-IV database. Restate the gap clearly at the beginning and end of the discussion and explain why this gap matters: Why would clinicians or researchers care about bicarbonate monitoring in ARDS?

Reply: Thanks for your comment. We further elaborated on the clinical significance of our study in the discussion section.

One of its major strengths was that it is the first to investigate the association between baseline bicarbonate levels and short-term mortality risk in critically ill ARDS patients, using a large-scale cohort. Baseline bicarbonate level, as a potential prognostic biomarker for ARDS, holds dual clinical significance: on the one hand, lower bicarbonate levels reflect the metabolic acidosis and is associated with multi-organ dysfunction; on the other hand, lower bicarbonate levels can provide a reference for the stratification of mortality risk and help guide more effective clinical management of patients with ARDS in the ICU.

- Ensure that all important claims are properly cited. Why females? Why mild ARDS? Some speculation is offered (gender physiology, compensation), Add citations for statements.

Reply: Thanks for your comment.

First, as clarified in our initial response to the reviewers, the ARDS patients included in this study reflect the **real-world gender distribution** of the MIMIC-IV database.

Second, in subsequent subgroup analyses stratified by patient characteristics, we observed that the association between baseline bicarbonate levels and 28-day mortality risk was **more pronounced in female ARDS patients** compared to males. To interpret this gender-based disparity, we propose in the Discussion that: Females may exhibit **distinct physiological responses** to metabolic acidosis or acid-base regulatory mechanisms, potentially rendering them more vulnerable to the adverse effects of low bicarbonate levels, thereby increasing mortality risk.

Similarly, subgroup results indicated that the bicarbonate-mortality association was **stronger in patien**ts with higher CCI scores and milder ARDS severity. A higher CCI is indicative of multiple comorbidities, which further complicates the management of ARDS and places an additional burden on the body. Comorbidities with higher CCI scores, such as diabetes mellitus or kidney disease, tend to exacerbate the inflammatory state, which is further exacerbated by acidosis. We hypothesize that different outcomes between patients with mild and severe ARDS may be attributable to differences in their compensation mechanisms for acid-base balance. These findings underscore the importance of monitoring bicarbonate levels in patients with ARDS in the treatment of this disease, particularly in specific subgroups of patients, as this may help identify patient populations at higher risk of death and provide guidance for the development of more individualized treatment strategies. Of course, the association between baseline bicarbonate level and ARDS patients in terms of gender differences, multiple comorbidities, or conditions still needs more research to further explore the underlying physiological and pathological mechanisms.

Moreover, we have already supplemented the citation of relevant references in the manuscript.

- The Limitations section is thorough but could be more concise. Merge similar points (single-center study, limited generalizability, need for external validation) into a tighter paragraph. Acknowledge potential reverse causality: Lower bicarbonate could be a marker of severity rather than a direct cause. Discuss missing variables: For instance, lactate levels or detailed ventilator parameters could confound the bicarbonate-mortality relationship.

Reply：Thanks for your comment. We have integrated the limitations of the study into a more concise statement, and at the same time, we have also discussed the influence of the missing variables on the current study:

However, we must acknowledge that our study has some objective limitations. First, as an observational, retrospective cohort study based on the MIMIC-IV database, our findings cannot establish a causal relationship between baseline bicarbonate levels and 28-day mortality risk in ARDS patients. Lower bicarbonate levels may serve as a prognostic marker rather than an independent risk factor for short-term outcomes. Second, being a single-center study, our results inevitably suffer from limited generalizability due to population heterogeneity, institution-specific practices, and insufficient data diversity. Therefore, caution was warranted when extrapolating these findings to patients from different regions, ethnicities, socioeconomic backgrounds, or healthcare settings. Third, although we adjusted for numerous potential confounders, residual bias may persist due to unmeasured variables and inherent limitations in MIMIC-IV data availability. Fourth, bicarbonate measurements were only available for ICU-admitted patients, restricting our ability to assess how post-ICU fluctuations in bicarbonate levels might influence mortality. This could affect the precision of our conclusions. Considering these limitations, further validation through well-designed multicenter prospective studies or randomized controlled trials (RCTs) is essential to confirm these associations and evaluate their broader clinical applicability.

Minor typos: "forth" (should be "fourth") at line 377.

Reply：Thanks for your comment. We have corrected the spelling mistake here.

- Ensure all proper nouns and terms (e.g., ARDS, ICU) are consistently capitalized.

Reply：Thanks for your comment. We have changed all the proper nouns and terms in the manuscript to capitalization.

- Phrasing: "definitive establishment of causality relationship" to "definitive establishment of a causal relationship."

Reply：Thanks for your comment. We have revised this sentence.

- Some sentences are quite lengthy and could benefit from splitting for clarity. E.g., Line 358: "The patients’ mortality risk could be driven by several mechanisms…"

Reply：Thanks for your comment. We have read through the full manuscript and revised some of the overly lengthy statements, hoping that our article can be more concise and easier to understand.
