# Supplementary material for: Association between bicarbonate levels and mortality among acute respiratory distress syndrome patients: An analysis based on Medical Information Mart for Intensive Care database
Source: PLoS One. 2025 Jun 10;20(6):e0325498. doi: 10.1371/journal.pone.0325498 (PMC12151428; doi:10.1371/journal.pone.0325498)
Supplement: Attachment — Submitted filename: Third responses to reviewers.docx [file pone.0325498.s005.docx]

**Methods:**

-Typo at line 137: "eGFR was categorized a 137 s <60..." → Should be: "eGFR was categorized as <60, 60–90, or ≥90 mL/min/1.73 m²."

Reply: Thanks for your comment. We have revised this typo.

-Line 114-115: Formatting issue with “, therefore,” and "Second Affiliated Hospital..."

Reply: Thanks for your comment. We have revised this typo.

Discussion: paragraph lines [349-361] needs supporting references.

Reply: Thans for your comment. We have added the supporting references for this section.
